# Efficacy Of Quadratus Lumborum Blocks (QLBs) in Robotic Nephrectomy: A Retrospective Study

**DOI:** 10.7759/cureus.36244

**Published:** 2023-03-16

**Authors:** Poonam Pai B.H., Abimbola Onayemi, Yan H Lai

**Affiliations:** 1 Anesthesiology, Mount Sinai Hospital, New York, USA

**Keywords:** enhanced recovery pathways, quadratus lumborum block, regional anesthesia, robotic laparoscopic nephrectomy, kidney surgeries

## Abstract

Introduction: Abdominal blocks such as quadratus lumborum block (QLB) have been used as an effective analgesic in abdominal surgeries. However, their efficacy in kidney surgery remains unknown. To the best of our knowledge, there are no clinical studies exploring the relationship between QLBs and post-operative opioid consumption in robotic laparoscopic nephrectomy.

Objectives: To assess the analgesic efficacy of QLB and its impact on perioperative opioid consumption in robotic laparoscopic nephrectomy.

Materials and methods: A retrospective chart review was conducted by querying the electronic medical record system of a 2,200-bed tertiary academic hospital center in New York City. The primary measured outcome was postoperative morphine milligram equivalents (MME) consumption for the first 24 hours. Secondary outcomes include intra-operative MME as well as postoperative pain scores measured on a visual analogue scale (VAS) scale at 2, 6, 12, 18, and 24 hours postoperatively.

Results: The mean total postoperative MME in the posterior QLB (pQLB) group was 11 in the QLB group (interquartile range (IQR) 4, 18) and 15 in the control group (IQR 5.6, 28) . There was a significant reduction in intraoperative MME in the QLB group in comparison to the control group. This reduction was not seen in postoperative MME. There was no significant difference in pain scores at any of the measured time points up to 24 hours postoperatively.

Conclusion: Our study provides compelling support that ultrasound guided QLB significantly decreased intraoperative opioid requirements but did not have the same effect on postoperative opioid requirements following robotic kidney surgeries in the context of an enhanced recovery after surgery (ERAS) pathway.

## Introduction

Approaches to contemporary urologic procedures range from open incisions to minimally invasive or robotically-guided techniques [1]. Contributions to postoperative pain in patients undergoing these surgeries may include the size of incision, location of port sites, pelvic organ nociception, discomfort due to urinary catheter, or diaphragmatic irritation from residual pneumoperitoneum [2,3]. Inadequately controlled postoperative pain may have harmful physiologic and psychological consequences that potentially increases perioperative morbidity and mortality [4,5]. Poor control of acute post-operative pain is associated with a higher incidence of progression to chronic pain [6]. Excellent pain control is a cornerstone of enhanced recovery after surgery (ERAS) pathways. Specifically, multimodal analgesia after renal surgeries has yielded beneficial perioperative outcomes such as improved functional recovery, reduced incidence of postoperative nausea and vomiting (PONV) and mitigation of adverse effects of opioids, all culminating in shorter hospital lengths of stay and increased patient satisfaction [7].

With varying success, regional anesthetic techniques, such as neuraxial anesthesia, transverse abdominis plane (TAP) blocks, and lumbar plexus blocks, have been employed as part of ERAS protocols for renal surgeries. Similarly, systemic opioids, nonsteroidal antiinflammatory drugs (NSAIDS), magnesium and lidocaine have also been studied [8]. The posterior quadratus lumborum block (pQLB) is an abdominal truncal block first described by Blanco in 2007 [9]. He characterized it as an ultrasound-guided posterior TAP block [9]. Anatomically, pQLB involves the injection of local anesthetic (LA) into a contiguous TAP interfascial plane that extends posteriorly to the thoracolumbar fascia (TLF) investing the quadratus lumborum muscle superiorly. The analgesic effect is produced by LA spread along these fascial planes targeting intercostal nerves that provide sensory innervation to the abdominal wall. Potential spreading patterns in cephalo-caudad as well as antero-posterior directions have been described, with potential involvement of paravertebral or neuraxial spaces [8].

Three types of QLB have been described and have anatomical names which describe the location of LA deposition around the QL muscle: QL1 or lateral, QL2 or posterior, and QL3 or transmuscular. Other modifications such as the paramedian sagittal oblique (PSO) and transverse oblique paramedian (TOP) orientations also exist [10]. These types of QLBs add complexity and diversity, but uncertainty to the efficacy of QLB in general. Based on the available literature on efficacy, ease of block placement, and complication profiles, we have adopted the pQLB as part of our ERAS protocol for robotic nephrectomies.

Previous studies have shown that the addition of pQLB to a regimen of morphine and NSAIDs is associated with a significant reduction in perioperative opioid consumption in abdominal surgeries [11-13]. However, the efficacy of pQLB in renal surgeries remains unknown. To our best knowledge, there are no clinical studies exploring the relationship between pQLB and post-operative opioid consumption in robotic laparoscopic nephrectomy.

This article has been previously presented at American Society of Regional Anesthesia (ASRA) conference, Las Vegas, in 2019.

This article was previously posted to research square on July 13, 2021.

## Materials and methods

A waiver of informed consent for retrospective chart review was obtained from Mount Sinai Institutional Review Board. All methods were performed in accordance with the relevant guidelines and regulations along with ethical approval and consent to participate as per the Declaration of Helsinksi. We included all patients who underwent robotic and laparoscopic kidney surgeries between January 2015 and May 2018 in a 2,200-bed academic medical center. The anesthesia information management system, Compurecord (Philips Medical, Andover, USA), electronic medical record PRISM (GE Healthcare, Chicago, USA) and EPIC (Epic Systems Corporation, Verona, USA) were queried. Intraoperative opioid administration, postoperative opioid consumption, information about blocks performed, and pain scores over the first postoperative 24 hours were collected.

All patients were monitored using standard American Society of Anesthesiology (ASA) monitors and neuromuscular blockade was administered to facilitate endotracheal intubation after anesthetic induction. All anesthetics were maintained with inhaled agents. All patients received opioids intraoperatively at the discretion of the anesthesia practitioner depending on the intraoperative mean arterial pressure (MAP) and heart rate. 

Patients were positioned in lateral decubitus position with operative side up. pQLB was performed under direct visualization with an ultrasound machine (Sonosite Inc., Bothell, USA) using a 13-6 MHz broadband curvilinear probe. Blocks were performed by an attending anesthesiologist or by a resident or a fellow under direct supervision, either prior to surgical incision or after the dressing was applied at the end of surgery. Blocks were placed with an insulated 22-gauge 50 mm block needle (Pajunk, Geisingen, Germany) inserted in an in-plane medial to lateral approach. After negative aspiration, 30 ml of long-acting LA (0.25% bupivacaine or 0.2% ropivacaine) was injected in 5 ml aliquots, with direct visualization of spread in the intersection of pQLB and the TAP fascial planes.

Surgery was performed by two surgeons at the hospital locations, all specializing in robotic guided laparoscopic nephrectomies. We excluded open procedures or surgeries that were converted to open incisions. We also excluded patients who were administered patient-controlled analgesia (PCA) postoperatively or patients who received other forms of regional anesthetics.

The surgical incisions for robotic guided laparoscopic partial nephrectomy involved three to four keyhole incisions each measuring 1 cm in the abdomen. All patients were expected to remain in the hospital for 48 hours after surgery.

Outcomes

The primary measured outcome was postoperative morphine milligram equivalent (MME) consumption for the first 24 hours. Secondary outcomes include intraoperative MME, as well as postoperative pain scores measured on a visual analogue scale (VAS) at 2, 6, 12, 18, and 24 hours postoperatively. Intraoperative MME was calculated based on total intraoperative consumption of fentanyl, hydromorphone, and morphine. Postoperative MME was calculated based on total administration of fentanyl, hydromorphone, morphine and oxycodone. Perioperative consumption of acetaminophen, remifentanil, ketamine, gabapentin, and ketorolac was also calculated. 

Intraoperative maintenance anesthetic either volatile anesthetic or total intravenous anesthesia (TIVA) after endotracheal intubation was recorded. 

Statistical analysis

Descriptive data are reported as a number (%), mean (± standard deviation) or median (interquartile range (IQR)). For group comparisons, two-sample t-test or the Wilcoxon-Mann-Whitney tests was used for continuous data, and Chi-square or Fisher Exact test was used for categorical data, as appropriate. Linear regression models were built to compare the log-transformed intraoperative and postoperative MME use between the pQLB and control groups. Covariates such as age, gender, ASA status, diabetes, hypertension, anesthesia team (resident or certified registered nurse anesthetist (CRNA)), anesthesia method (volatile anesthetic or TIVA), surgical duration, and hospital site were adjusted between the two groups. 

Logistic regression model using the generalized estimating equations method was used to compare the incidence of severe pain (pain score ≥ 7) between the two groups during the first 24-hour period following surgery, and to test whether this incidence difference was the same over time. Odds ratios of reporting severe pain (VAS of more than 7) at 2, 6, 12, 18 and 24 hours postoperatively and the corresponding confidence intervals derived from the empirical standard errors were recorded. Analysis was performed using SAS 9.4 (SAS Institute Inc., Cary, USA). All tests were two-sided and statistical significance was defined as a p value < 0.05, unless otherwise specified.

## Results

Out of the total 722 patients identified, 148 were excluded based on predetermined criterion. 211 of the 554 patients who underwent laparoscopic/robotic-assisted partial nephrectomies received a pQLB as part of their analgesic strategy. The rest that did not receive QL block were in the other group of 343 patients. Demographic data appears in Table 1. There was no significant difference observed between groups. Blocks were administered prior to surgical incision in 90.52% (191) and after closure in 9.48% (20).

**Table 1:** Demographic variables pQLB: Posterior quadratus lumborum block; CRNA: Certified registered nurse anesthetist; MSH: Mount Sinai Hospital; MSW: Mount Sinai West; ASA: American Society of Anesthesiology; TIVA: Total intravenous anesthesia

Variables			pQLB (n=211)	NO pQLB (n=343)	P value
Age (in Years)			60 ± 13	59 ± 12	0.5
Sex (Male/Female)			75/136	136/207	0.3
Diabetes			34 (16%)	64 (18%)	0.4
Hypertension			115 (54.5%)	214 (62.4%)	0.05
Smoking Status (Yes/No)			19 (11.1%)	51 (14.8%)	0.04
Anesthesia Team		Resident	200 (94.7%)	259 (75.5%)	0.01
		CRNA	11 (0.03%)	84 (24.5%)	
Hospital Site		MSH	21 (10%)	65 (19%)	0.005
		MSW	190 (90%)	278 (81%)	
Anesthesia Type		General anesthesia with TIVA	173	17	0.001
		General anesthesia with volatile	38	326	
ASA Status		1	8	11	0.07
		2	126	168	
		3	67	139	
		4	10	25	
Surgery Duration (min)			136 (118, 161)	151 (120, 182)	0.001
Type of Surgery	Laparoscopic		127 (60%)	233 (68%)	0.001
	Robotic		84 (40%)	110 (32%)	
	Partial		121 (57.3%)	229 (66.7%)	
	Radical		90 (42.6%)	114 (33.2%)	
Block Timing		Before Incision	191 (90%)	-	0.001
		After Surgery	20 (10%)	-	
Local Anesthesia		Bupivacaine	202 (95.8%)	-	0.001
		Ropivacaine	9 (4.2%)	-	

The median surgical duration was 136 minutes in the pQLB group (IQR 118, 161) and 151 minutes in the control group (IQR 120, 182) (p = 0.001). The median total intraoperative MME in the group receiving the pQLB was 10 (3, 25), compared to 55 (40, 85) in the group that did not receive the block (p = 0.001). Specifically, total intraoperative fentanyl administration was significantly reduced in the pQLB group (p = 0.007). The mean total postoperative MME in the pQLB group was 11 in the pQLB group (4, 18) and 15 in the control group (5.6, 28) (Table 2). After covariate adjustment between the two groups, the reduction in postoperative MME in the pQLB group was not significant. 

**Table 2:** Intraoperative and postoperative variables pQLB: Posterior quadratus lumborum block; IQR: Interquartile range; MME: Morphine milligram equivalent

VARIABLES	pQLB (n=211)			NO pQLB (n=343)		P value
Intraoperative analgesics	Number of patients	Median (IQR)		Number of patients	Median (IQR)	
Ketamine (in mg)	108	140 (100, 151)		14	124 (88, 156)	0.3
Remifentanil (in mg)	4	1.8 (1.4, 2.1)		23	1.2 (0.9, 2.0)	0.2
Acetaminophen (in mg)	128	1,000 (1,000, 1,000)		109	1,000 (1,000, 1,000)	0.4
Ketorolac (in mg)	4	30 (30, 30)		13	30 (30, 30)	0.7
Fentanyl (in mcg)	211	100 (0, 250)		343	500 (300, 600)	<0.001
Hydromorphone (in mg)	211	0 (0, 0)		343	0 (0, 1)	<0.001
Morphine (in mg)	0	0 (0, 0)		0	0 (0, 0)	1
Total intraoperative MME	211		10 (3, 25)	343	55 (40, 85)	<0.001
Postoperative analgesics:						
Acetaminophen (in mg)	26		1,300 (650, 1300)	65	975 (650, 1,300)	0.35
Fentanyl (in mcg)	117		100 (75, 100)	192	100 (75, 150)	0.007
Hydromorphone (in mg)	108		1 (0.75, 1.5)	155	1.5 (1, 2.5)	0.001
Oxycodone (in mg)	134		12.5 (10, 30)	200	20 (10, 30)	<0.001
Morphine (in mg)	31		4 (2, 6)	100	8 (4, 12)	<0.001
Gabapentin (in mg)	3		300 (100, 800)	6	450 (300, 600)	0.79
Ketorolac (in mg)	12	53 (30, 60)		39	60 (45, 90)	0.10
Total postoperative MME	211	11 (4, 18)		343	15 (5.6, 28)	0.001

The median pain scores at various time points (2, 6, 12, 18, 24 hours) ranged from 2 (IQR 0-5) to 4 (IQR 0-6) for the pQLB group, and 2 (IQR 0-5) to 3 (IQR 0-5) in the control group (p value>0.05) (Table 3). Additionally, there was a trend towards greater reduction of intraoperative MME when the block was placed after surgery (Table 4). There was no significant difference in pain scores during the first 24 hours between the two groups. However, the odds of having severe pain tended to be consistently higher in the pQLB group. 

**Table 3:** Pain scores * Wilcoxon rank sum test Pain scores at Q2, Q6, Q12, Q18 and Q24 in medial and IQR with p value. pQLB: Posterior quadratus lumborum block; IQR: Interquartile range

Time Interval	pQLB (n=211)			No pQLB (n=343)			P value*
	Minimum	Maximum	Median (IQR)	Minimum	Maximum	Median (IQR)	
Q2	0	10	4 (0, 6)	0	10	3 (0, 5)	0.06
Q6	0	10	2 (0, 5)	0	10	3 (0, 5)	0.21
Q12	0	10	2 (0, 5)	0	10	3 (0, 5)	1
Q18	0	10	3 (0, 5)	0	10	3 (0, 5)	0.64
Q24	0	10	3 (0, 5)	0	10	2 (0, 5)	0.07

 

**Table 4:** Effect of QLB on MME during and after surgery * Based on linear models adjusting for age, gender, diabetes, hypertension comorbidities, ASA status, anesthesia team, anesthesia method, surgical duration and hospital site. QLB: Quadratus lumborum block; MME: Morphine milligram equivalent; ASA: American Society of Anesthesiology

Table 4: Effect of QLB on MME during and after surgery						
Period	QL block	Variables	*Logarithmic scale of MME Difference	Standard Error	p-value	Overall p-value
Intraoperative	Overall	Yes	-0.63	0.30	0.033	0.033
		No	(ref)			
	Block timing	After surgery	-1.02	0.50	0.042	0.065
		Before incision	-0.54	0.31	0.079	
		Not done	(ref)			
Postoperative	Overall	Yes	-0.27	0.38	0.480	0.480
		No	(ref)			
	Block timing	After surgery	-0.85	0.64	0.184	0.411
		Before incision	-0.14	0.39	0.727	
		Not done	(ref)			

## Discussion

This is a retrospective review of patients who underwent robotic and laparoscopic kidney surgeries. There was a significant reduction in intraoperative MME in the group of patients who received a pQLB in comparison to the control group. This reduction was not seen in postoperative MME, nor was there any significant difference in pain scores at any of the measured time points up to 24 hours postoperatively.

Managing postoperative pain in kidney surgeries presents a unique set of challenges. In our multimodal analgesic and ERAS pathways, avoidance of NSAIDs and opioids is prudent owing to concerns of nephrotoxicity with accumulation of active and nondialyzable metabolites [12]. The possibility of impaired creatinine clearance must be considered when dosing opioids as well. With the increasing numbers of kidney surgeries occurring worldwide, further exploration of modern analgesic techniques is timely [14].

Ultrasound-guided regional anesthesia is highly beneficial after abdominal surgery. Truncal blocks such as TAP blocks have been performed in donor nephrectomies and were found to result in reduced pain scores and reduced morphine consumption during the first 24 hours postoperatively [14,15]. Li et al. performed a placebo controlled randomized control trial in partial and radical nephrectomy and found that preoperative TAP block was effective in reducing intraoperative and postoperative opioid consumption but had no effect on pain scores and length of stay [16]. The pQLB, a variation of the posterior TAP block, is being studied extensively in abdominal surgeries. Blanco et al. compared TAP blocks to pQLBs after Caesarean section under spinal anesthesia [17]. They concluded that pQLB significantly reduced the consumption of morphine after cesarean delivery in comparison to TAP blocks [17].

However, the efficacy of pQLB in kidney surgeries remains heterogeneously ambiguous. Elsharkawy et al. reported that the use of transmuscular QLB provided appropriate sensory blockade for open urological surgeries [18]. Using a pQLB approach, Zhu et al. concluded that patients in the pQLB group had lower sufentanil consumption in the first 24 hours after laparoscopic nephrectomy when compared to the control group [19]. In contrast, Boulianne et al. did not observe a reduction in postoperative opioid administration at 24 hours with a pQLB in patients undergoing elective colorectal surgery. Post-operative pain scores were actually lower in the control group [20].

Although QLBs are widely adopted into practice despite firm evidence on their efficacy, complications, and nerve targets or LA spreading patterns, there have been some mixed results regarding effectiveness of pQLB. Various authors conclude that it is important to consider the type of pQLB while interpreting study results. Kukreja et al. demonstrated that the VAS pain score at 24 hours and cumulative opioid consumption were significantly lower in the anterior QLB group after total hip arthroplasty (THA) as compared with controls [21]. Without a pQLB comparison group, Green et al. suggested that the use of transmuscular QLB in patients undergoing THA shortened the length of stay to an average of 2.9 days in comparison to 5.1 days. The intraoperative fentanyl use was also considerably lower in the block group under general anesthesia [22]. In major orthopedic procedures, Hebl et al. showed that ERAS programs utilizing peripheral nerve blocks had significant impacts on perioperative outcomes, such as shortened hospital length of stay and fewer adverse events such as ileus and urinary retention [23]. Patients participating in ERAS pathways had lower postoperative pain scores, opioid requirements, and incidences of adverse effects.

The timing of the performance (pre or postoperatively) of the pQLB is an important factor that is rarely studied. The pre-incision pQLB timing in our study was not associated with reduction of total MME for both the intraoperative and postoperative periods, with the odds ratio of having lower pain scores if the block was performed after the conclusion of surgery. Surgical incision triggers an inflammatory reaction to damaged tissues that induces central sensitization of pain pathways. It has been proposed that anti­nociceptive protection provided by preemptive treatments should extend into the postoperative period to effectively cover the inflammatory phase. In turn, preemptive analgesia focuses on minimizing central sensitization and potentially decreasing the incidence of developing chronic neuropathic pain [24]. Similar to our study, Olanipekun et al. conducted a study comparing preincisional and postincisional ilioinguinal and iliohypogastric nerve block to control postoperative pain in children after herniotomy. They concluded that though the mean pain scores were lower in the preincision group at six hours, they were higher at 24 hours in comparison to the postoperative block group [25].

Our study has limitations that are inherent to a retrospective study. Anesthetic practices and regional anesthetic techniques were neither controlled nor standardized. Thus, the true efficacy and effectiveness of pQLB could be underreported. Also, some patients might have been on chronic opioids and have had higher tolerance to pain medications. Due to the retrospective nature of the study, some of the confounding variables could not be accounted for. Randomized controlled trials assessing the efficacy should focus on standardization of techniques including the types of QLB utilized. The size of our surgical cohort was sufficient to demonstrate statistical significance. In spite of the pQLB being performed under general anesthesia, presence of non-blinded practitioners in the perioperative period could have introduced further bias due to decision-making in terms of opioid requirements in the pQLB group. 

## Conclusions

In conclusion, there was a significant reduction in intraoperative MME in the group of patients who received a pQLB in comparison to the control group. This reduction was not seen in postoperative MME, nor was there any significant difference in pain scores at any of the measured time points up to 24 hours postoperatively. Hence, our study provides compelling evidence that ultrasound-guided pQLB significantly decreases intraoperative, but not postoperative, opioid requirements for robotic kidney surgeries in the context of an ERAS pathway.

## References

[1] Frampton S, Kneebone RL (2017). John Wickham's new surgery: 'minimally invasive therapy', innovation, and approaches to medical practice in twentieth-century Britain. Soc Hist Med.

[2] Hager B, Herzog SA, Hager B, Sandner-Kiesling A, Zigeuner R, Pummer K (2019). Comparison of early postoperative pain after partial tumour nephrectomy by flank, transabdominal or laparoscopic access. Br J Pain.

[3] Mathuram Thiyagarajan U, Bagul A, Nicholson ML (2012). Pain management in laparoscopic donor nephrectomy: a review. Pain Res Treat.

[4] Gan TJ (2017). Poorly controlled postoperative pain: prevalence, consequences, and prevention. J Pain Res.

[5] Joshi GP (2005). Multimodal analgesia techniques and postoperative rehabilitation. Anesthesiol Clin North Am.

[6] Katz J, Jackson M, Kavanagh BP, Sandler AN (1996). Acute pain after thoracic surgery predicts long-term post-thoracotomy pain. Clin J Pain.

[7] Batley SE, Prasad V, Vasdev N, Mohan-S G (2016). Post-operative pain management in patients undergoing robotic urological surgery. Curr Urol.

[8] Gelman D, Gelmanas A, Urbanaitė D (2018). Role of multimodal analgesia in the evolving enhanced recovery after surgery pathways. Medicina (Kaunas).

[9] Abrahams M, Derby R, Horn JL (2016). Update on ultrasound for truncal blocks: a review of the evidence. Reg Anesth Pain Med.

[10] Balocco AL, López AM, Kesteloot C (2021). Quadratus lumborum block: an imaging study of three approaches. Reg Anesth Pain Med.

[11] Ueshima H, Otake H, Lin JA (2017). Ultrasound-guided quadratus lumborum block: an updated review of anatomy and techniques. Biomed Res Int.

[12] Akerman M, Pejčić N, Veličković I (2018). A review of the quadratus lumborum block and ERAS. Front Med (Lausanne).

[13] Blanco R, Ansari T, Girgis E (2015). Quadratus lumborum block for postoperative pain after caesarean section: a randomised controlled trial. Eur J Anaesthesiol.

[14] Gopwani SR, Rosenblatt MA (2016). Transversus abdominis plane block in renal allotransplant recipients: a retrospective chart review. Saudi J Anaesth.

[15] Aniskevich S, Taner CB, Perry DK (2014). Ultrasound-guided transversus abdominis plane blocks for patients undergoing laparoscopic hand-assisted nephrectomy: a randomized, placebo-controlled trial. Local Reg Anesth.

[16] Li X, Xu ZZ, Li XY, Jiang TT, Lin ZM, Wang DX (2019). The analgesic efficacy of ultrasound-guided transversus abdominis plane block for retroperitoneoscopic renal surgery: a randomized controlled study. BMC Anesthesiol.

[17] Blanco R, Ansari T, Riad W, Shetty N (2016). Quadratus lumborum block versus transversus abdominis plane block for postoperative pain after cesarean delivery: a randomized controlled trial. Reg Anesth Pain Med.

[18] Elsharkawy H, Ahuja S, DeGrande S, Maheshwari K, Chan V (2019). Subcostal approach to anterior quadratus lumborum block for pain control following open urological procedures. J Anesth.

[19] Zhu M, Qi Y, He H, Lou J, Pei Q, Mei Y (2019). Analgesic effect of the ultrasound-guided subcostal approach to transmuscular quadratus lumborum block in patients undergoing laparoscopic nephrectomy: a randomized controlled trial. BMC Anesthesiol.

[20] Boulianne M, Paquet P, Veilleux R (2020). Effects of quadratus lumborum block regional anesthesia on postoperative pain after colorectal resection: a randomized controlled trial. Surg Endosc.

[21] Kukreja P, MacBeth L, Sturdivant A, Morgan CJ, Ghanem E, Kalagara H, Chan VW (2019). Anterior quadratus lumborum block analgesia for total hip arthroplasty: a randomized, controlled study. Reg Anesth Pain Med.

[22] Stuart Green M, Ryan Hoffman C, Iqbal U, Olabisi Ives O, Hurd B (2018). Transmuscular quadratus lumborum block reduces length of stay in patients receiving total hip arthroplasty. Anesth Pain Med.

[23] Hebl J, Dilger J, Byer D (2008). A Pre-Emptive Multimodal Pathway Featuring Peripheral Nerve Block Improves Perioperative Outcomes After Major Orthopedic Surgery.. Reg Anesth Pain Med.

[24] Price TJ, Inyang KE (2015). Commonalities between pain and memory mechanisms and their meaning for understanding chronic pain. Prog Mol Biol Transl Sci.

[25] Olanipekun SO, Adekola OO, Desalu I, Kushimo OT (2015). The effect of pre-incision field block versus post-incision inguinal wound infiltration on postoperative pain after paediatric herniotomy. Open Access Maced J Med Sci.

